# Retrograde open celiac stenting for ischemic hepatitis after pancreaticoduodenectomy

**DOI:** 10.1016/j.jvscit.2023.101136

**Published:** 2023-03-04

**Authors:** Roberto G. Aru, Sarah E. Deery, Yasaman Kavousi, James H. Black, William R. Burns, Caitlin W. Hicks

**Affiliations:** a Division of Vascular Surgery and Endovascular Therapy, Johns Hopkins University School of Medicine, Baltimore, MD; b Department of Surgery, Johns Hopkins University School of Medicine, Baltimore, MD; c Division of Vascular and Endovascular Surgery, Maine Medical Center, Portland, ME; d Division of Vascular Surgery, Henry Ford Hospital, Detroit, MI; e Division of Surgical Oncology, Johns Hopkins University School of Medicine, Baltimore, MD

**Keywords:** Celiac artery revascularization, Ischemic hepatitis, Pancreaticoduodenectomy, Retrograde open mesenteric stenting, Whipple procedure

## Abstract

A 74-year-old man with pancreatic cancer had undergone pancreaticoduodenectomy and subsequently developed ischemic hepatitis secondary to high-grade celiac artery stenosis. Celiac antegrade stenting via brachial artery access was unsuccessful, and open antegrade bypass would have required takedown of the pancreatic and/or biliary anastomoses for adequate exposure. Retrograde open celiac stenting was, therefore, successfully performed via the gastroduodenal artery stump. His ischemic hepatitis resolved, and he was ultimately discharged with dual antiplatelet therapy. Computed tomography angiography at 6 months demonstrated a widely patent celiac stent. Retrograde open celiac stenting via the gastroduodenal artery stump is an alternative to open bypass for celiac revascularization not amenable to percutaneous antegrade stenting in patients who have undergone pancreaticoduodenectomy.

## Case report

A 74-year-old man with a remote history of prostate cancer status after prostatectomy in 2009 and non-Hodgkin lymphoma after chemotherapy in 2010 had presented with obstructive jaundice due to a mass in the pancreatic head. Endoscopic retrograde cholangiopancreatography with common bile duct stenting was performed, and an endoscopic ultrasound-guided biopsy confirmed adenocarcinoma. The patient's tumor was localized to the pancreas without evidence of metastasis. The genetic workup identified a germline BRCA2 mutation. He received neoadjuvant chemotherapy and was considered for curative intent surgery. Preoperative computed tomography angiography (CTA) demonstrated a good tumor response with a decrease in the head of the pancreas mass and chronic moderate (50%) stenosis of the celiac artery ostium. His superior mesenteric artery (SMA) was widely patent without significant disease. The patient provided written informed consent for the report of his case details and imaging studies.

Diagnostic laparoscopy did not identify occult metastasis; thus, the patient underwent laparotomy and pancreaticoduodenectomy with en bloc resection of the portomesenteric confluence with primary reconstruction, resulting in <50% stenosis of the portal vein. Before resection, test clamping of the gastroduodenal artery (GDA) yielded a diminished hepatic pulse, prompting median arcuate ligament release by the surgical oncologist to relieve extrinsic compression, with reported improvement in hepatic flow shown by Doppler ultrasound interrogation.

**Fig 1:**
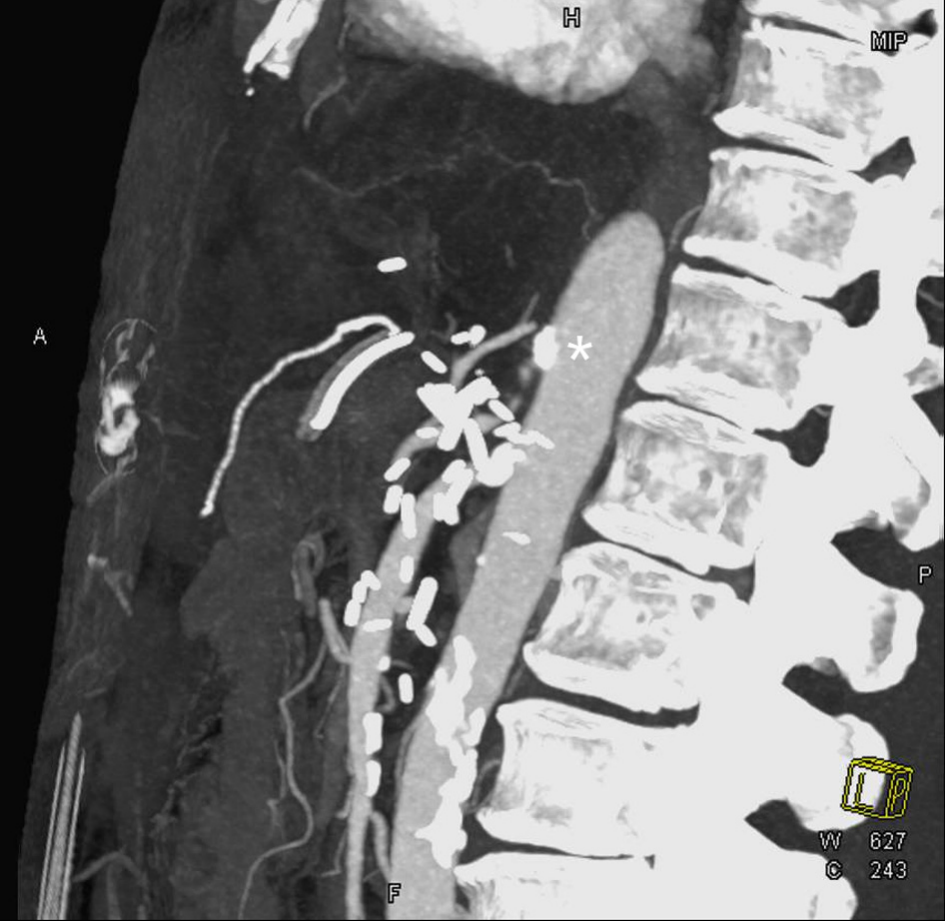
Sagittal image of computed tomography angiography (CTA) of the abdomen and pelvis demonstrating occlusion of the celiac artery (*asterisk*) and a patent superior mesenteric artery (SMA).

**Fig 2:**
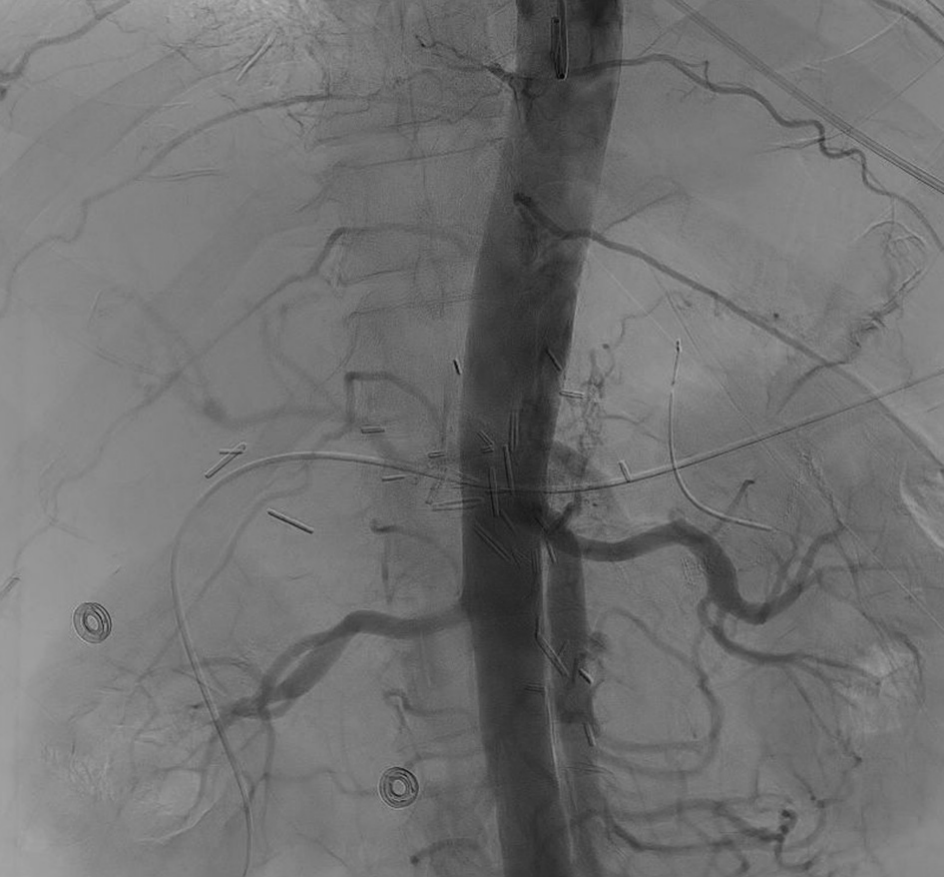
Diagnostic aortogram showing an occluded celiac artery.

In the postoperative period, the patient developed ischemic hepatitis with significantly elevated transaminases (peak alanine transaminase, 3150 U/L; aspartate transaminase, 1862 U/L). Interval CTA demonstrated new occlusion of the celiac artery (Fig 1), hypoperfusion of the left hepatic lobe, and <50% stenosis of the reconstructed portomesenteric confluence. No intraoperative hypotensive episodes or other causes of acute celiac artery thrombosis had occurred. Interventional radiology was unable to cannulate the occluded celiac artery (Fig 2) for antegrade celiac artery stent placement via percutaneous left brachial access.

**Fig 3:**
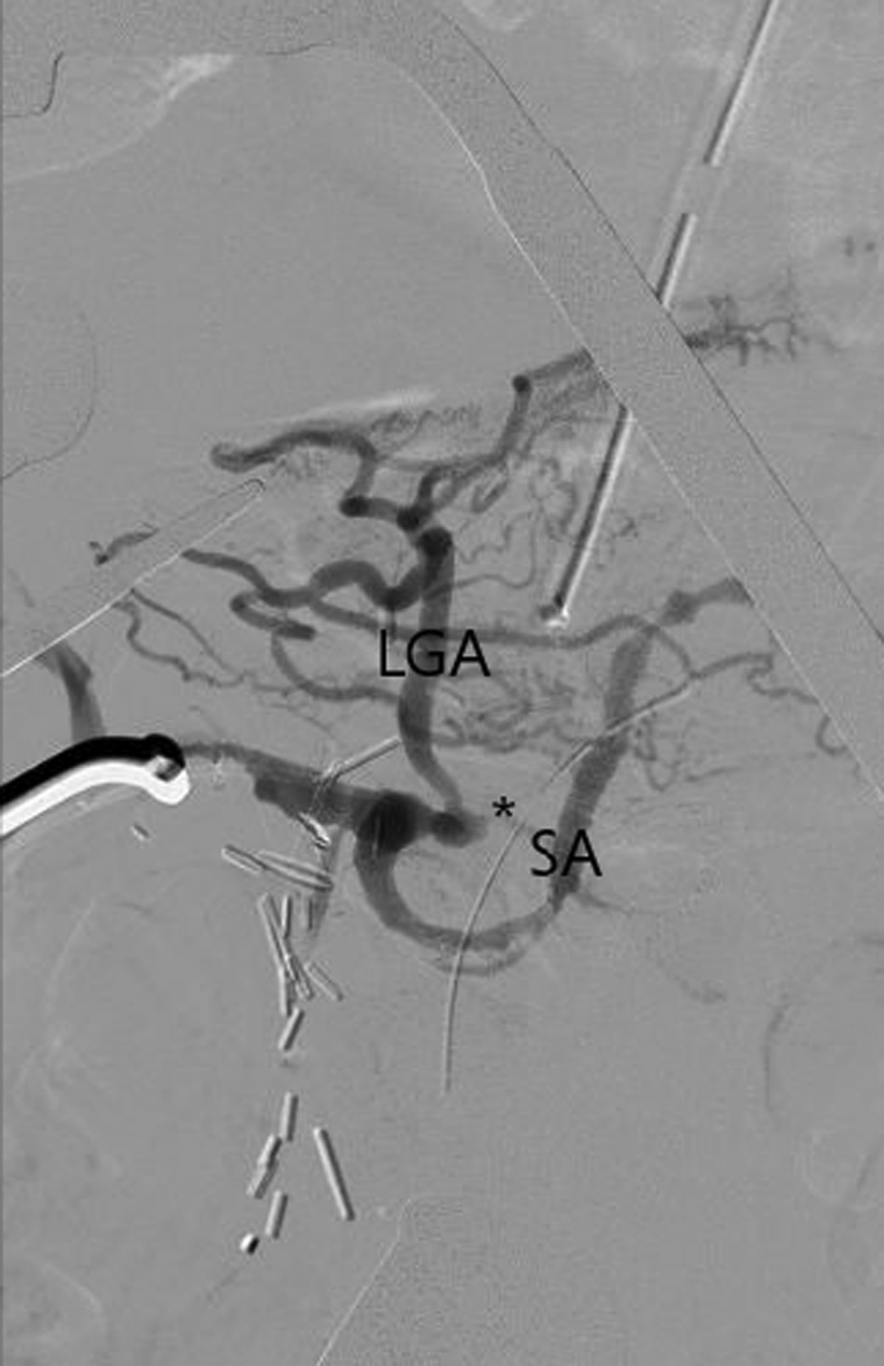
Diagnostic retrograde angiogram via gastroduodenal artery (GDA) micropuncture sheath showing an occluded celiac trunk (*asterisk*) and patent splenic artery (*SA*) and left gastric artery (*LGA*).

**Fig 4:**
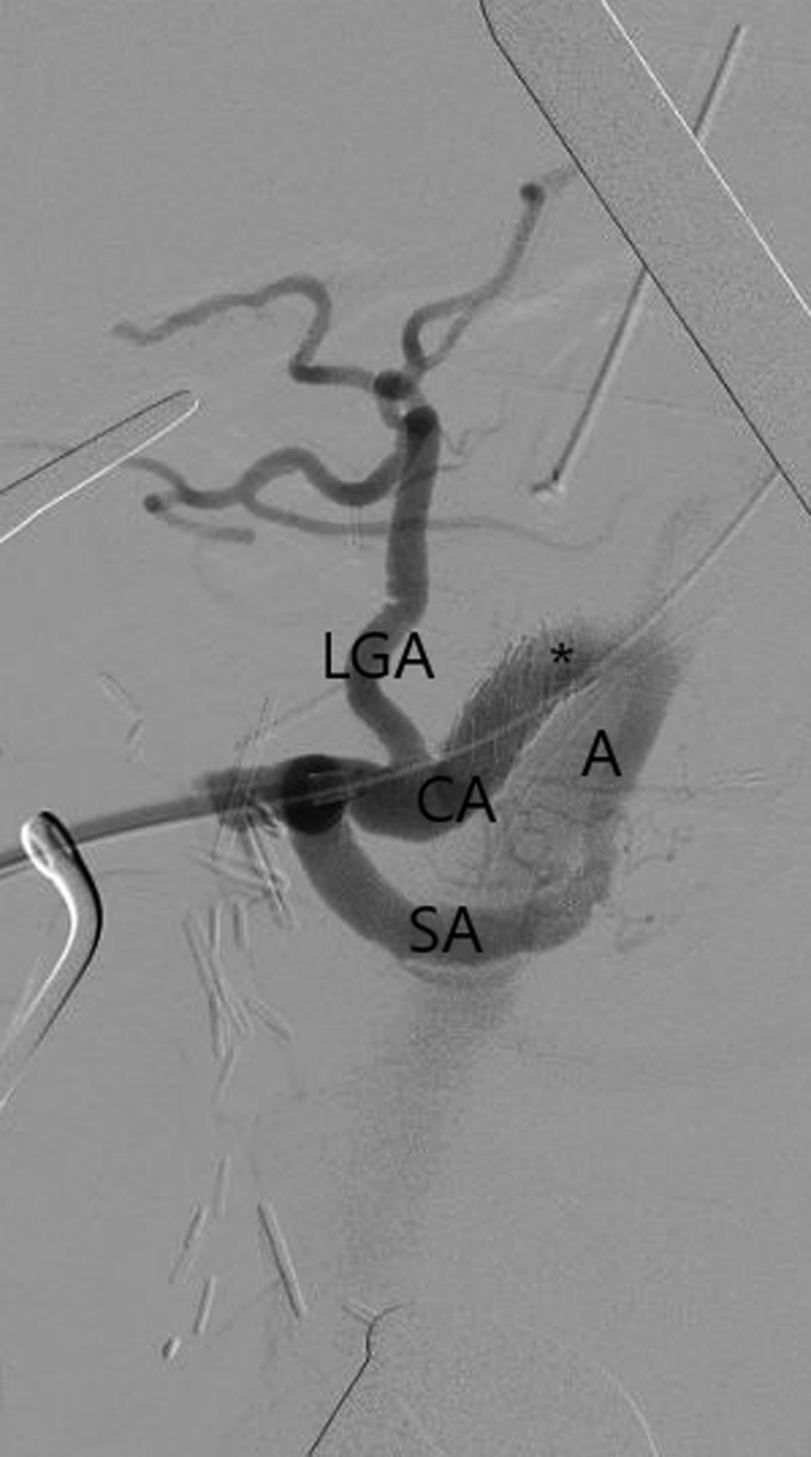
Completion angiogram showing robust flow via the celiac artery (*CA*) stent (*asterisk*) into the abdominal aorta (*A*). *LGA,* left gastric artery; *SA,* splenic artery.

Thus, vascular surgery was consulted, and the patient was taken to the hybrid operating room for emergent reopening of the recent laparotomy and hepatic artery revascularization. The decision was made to proceed with retrograde open celiac stenting instead of open antegrade bypass to avoid takedown of the biliary reconstruction and/or pancreatic reconstruction to achieve adequate exposure. The common hepatic artery was identified, dissected, and noted to be flaccid. After systemic heparinization, the ligated GDA stump was reopened, with minimal backbleeding. A diagnostic angiogram via a micropuncture sheath within the GDA demonstrated the takeoff of the splenic artery and left gastric artery without visualization of the proximal celiac artery (Fig 3). In the lateral view, an 0.018-in. crossing wire and catheter were used to traverse the celiac orifice into the abdominal aorta via a 7F sheath inserted directly into the GDA, and a short (7 × 19-mm), balloon-expandable covered stent was deployed in the proximal celiac artery, preserving the left gastric artery. If necessary, a bare metal stent would have been used to extend distally to preserve left gastric artery flow. A completion retrograde angiogram demonstrated excellent filling of the left gastric and splenic arteries, with restoration of flow through the celiac artery into the abdominal aorta (Fig 4). The sheath was removed, and the GDA stump was religated. New pulsatility was present within the common and proper hepatic arteries with biphasic signals on Doppler examination. The laparotomy was closed, and the patient was transferred back to the intensive care unit for observation.

**Fig 5:**
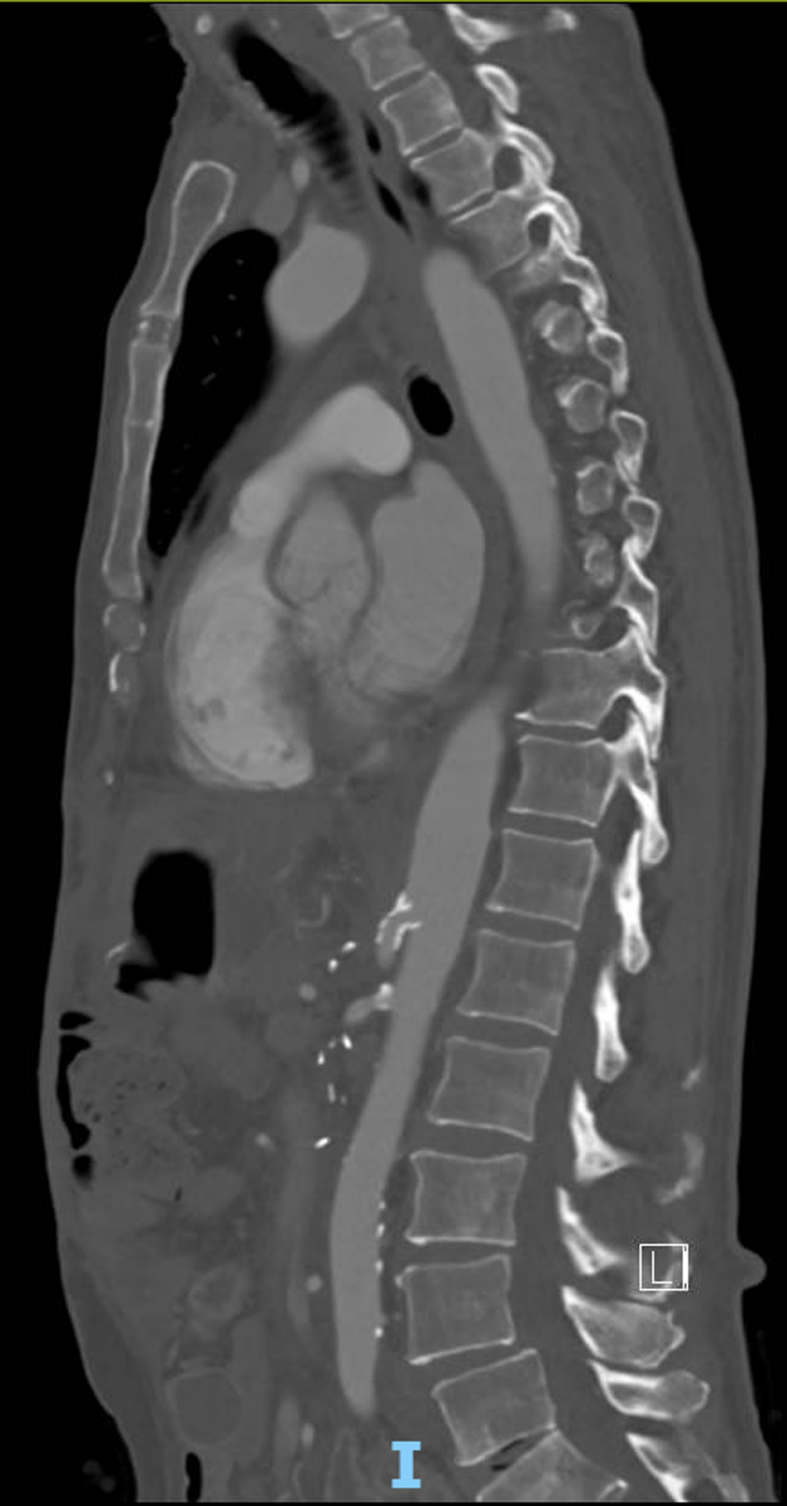
Computed tomography angiography (CTA) at 6 months postoperatively of the abdomen and pelvis showing a patent celiac artery stent extending proximally 1 to 2 mm into the abdominal aorta.

Rapid improvement occurred in the patient's liver function tests after revascularization, with normalization by postrevascularization day 3 (postoperative day 5 from the initial operation). His postoperative course was complicated by immune-related thrombocytopenia requiring intravenous immunoglobulin. He was ultimately discharged on postoperative day 16 with instructions to take aspirin 81 mg and clopidogrel (Plavix) 75 mg after his thrombocytopenia had resolved. The 6-week surveillance mesenteric duplex ultrasound demonstrated a patent celiac artery stent with appropriate flow velocities, and the 6-month postoperative CTA (Fig 5) confirmed these findings. His antiplatelet regimen was deescalated to aspirin monotherapy, and he resumed adjuvant chemotherapy. The patient remained without evidence of recurrent cancer at 6 months after his curative intent surgery.

## Discussion

Mesenteric atherosclerotic disease is increasingly common in an aging population. Intestinal perfusion is dependent on rich collateral networks between the mesenteric vessels. The incidence of celiac artery stenosis ranges from 10% to 25%.^1^ The predominant collateral between the celiac artery and SMA is the pancreaticoduodenal arcade, which will be interrupted by ligation of the GDA during pancreaticoduodenectomy. Thus, patients undergoing pancreaticoduodenectomy in the setting of celiac disease will be prone to postoperative liver failure or biliary stricture formation.^1^ In a retrospective, single-institution review, ischemic hepatitis occurred in 5 of 545 patients (0.9%) who had undergone pancreaticoduodenectomy. In addition, 5% of this cohort had had hemodynamically significant visceral stenoses based on selective visceral angiography that had been stented preoperatively (n = 3), bypassed intraoperatively (n = 1), or treated by median arcuate ligament division (n = 23).^2^ Revascularization of high-grade celiac stenoses should be performed before pancreaticoduodenectomy whenever possible to mitigate later ischemic complications.

Historically, open bypass was the preferred method for mesenteric revascularization. However, contemporary trends have favored an endovascular first approach, ranging from percutaneous stenting to retrograde open mesenteric stenting (ROMS). During the past 15 years, the popularity of hybrid stenting for SMA revascularization in the presence of acute and chronic mesenteric ischemia has increased.^3^, ^4^, ^5^, ^6^, ^7^, ^8^, ^9^, ^10^ In patients with acute mesenteric ischemia, an initial laparotomy will frequently be performed to assess intestinal viability. ROMS offsets the risk of supraceliac aortic exposure and graft infection associated with bypass. A number of institutional studies have reported excellent technical success and short- and mid-term outcomes for ROMS.^4^^,^^7^^,^^9^^,^^10^

Because the SMA will be preferentially revascularized to treat acute and chronic mesenteric ischemia, a paucity of data is available describing the ROMS technique for celiac artery revascularization. Rego et al^11^ described an elderly man with concomitant SMA occlusion and high-grade celiac stenosis who had undergone oncologic resection of a distal gastric cancer and had developed septic shock due to anastomotic dehiscence with subsequent open repair. In this low-flow state, acute mesenteric ischemia and ischemic hepatitis ensued, requiring repeat laparotomy, total abdominal colectomy, small bowel resection, and splenectomy. Retrograde open celiac artery stenting via the splenic artery stump restored hepatic arterial perfusion without additional morbidity. Their case was the first to extrapolate from the extensive experience in the literature on ROMS for SMA revascularization to celiac artery recanalization.

Celiac artery revascularization unamenable to percutaneous stenting is a unique clinical scenario in patients after pancreaticoduodenectomy. Supraceliac aorta to hepatic artery antegrade bypass is a well-described technique^12^; however, the pancreaticojejunostomy and hepaticojejunostomy anastomoses will limit the exposure for bypass. Likewise, the potential exists, albeit low, for the risk of graft infection similar to that with renohepatic bypass. Retrograde celiac artery stenting (ROCS) provides an alternative method for celiac artery revascularization. The GDA stump will be ligated as part of the pancreaticoduodenectomy and provides a viable option for sheath placement while minimizing the risks of access-related complications to the common hepatic artery. In addition to intraluminal recanalization, a technical challenge to this approach is a celiac trunk with enough length to accommodate a balloon-expandable stent graft without sacrificing the visceral branches such as the left gastric artery, which is important for gastric perfusion after pancreaticoduodenectomy. If these obstacles can be overcome, ROCS via the GDA stump can provide an elegant solution for celiac artery revascularization. This technique is most widely applicable using a mobile C-arm at the time of pancreaticoduodenectomy when dampening or loss of the hepatic artery pulse occurs during GDA test clamping as a prophylactic intervention.

## Conclusions

Similar to ROMS for SMA revascularization, ROCS is a viable alternative to percutaneous stenting and bypass for celiac artery lesions. For patients who have undergone pancreaticoduodenectomy, the GDA stump provides a safe retrograde access point to the celiac artery for hybrid stenting.
